# Why do people purchase antibiotics over-the-counter? A qualitative study with patients, clinicians and dispensers in central, eastern and western Nepal

**DOI:** 10.1136/bmjgh-2021-005829

**Published:** 2021-05-11

**Authors:** Bipin Adhikari, Sunil Pokharel, Shristi Raut, Janak Adhikari, Suman Thapa, Kumar Paudel, Narayan G C, Sandesh Neupane, Sanjeev Raj Neupane, Rakesh Yadav, Sirapa Shrestha, Komal Raj Rijal, Sujan B Marahatta, Phaik Yeong Cheah, Christopher Pell

**Affiliations:** 1 Mahidol Oxford Tropical Medicine Research Unit, Faculty of Tropical Medicine, Mahidol University, Bangkok, Thailand; 2 Centre for Tropical Medicine and Global Health, Nuffield Department of Medicine, University of Oxford, Oxford, UK; 3 Universal College of Medical Sciences and Teaching Hospital, Bhairahawa, Nepal; 4 BP Koirala Institute of Health Sciences, Dharan, Nepal; 5 Patan Academy of Health Sciences, Patan, Nepal; 6 Country Coordinating Mechanism, The Global Fund, Ministry of Health and Population, Kathmandu, Nepal; 7 Australian Federation of AIDS Organisations, Bangkok, Thailand; 8 Nepal Public Health Research & Development Centre, Kathmandu, Nepal; 9 Manmohan Memorial Institute of Health Sciences, Kathmandu, Nepal; 10 Central Department of Microbiology, Tribhuvan University, Kirtipur, Nepal; 11 Centre for Social Science and Global Health, University of Amsterdam, Amsterdam, The Netherlands; 12 Amsterdam Institute for Global Health and Development, Amsterdam, The Netherlands

**Keywords:** qualitative study, infections, diseases, disorders, injuries, health policy, health services research, health systems

## Abstract

**Introduction:**

Over-the-counter (OTC) use of antibiotics contributes to the burgeoning rise in antimicrobial resistance (AMR). Drawing on qualitative research methods, this article explores the characteristics of OTC sales of antibiotic in Nepal, its drivers and implications for policy.

**Methods:**

Data were collected in and around three tertiary hospitals in eastern, western and central Nepal. Using pre-defined guides, a mix of semi-structured interviews and focus group discussions were conducted with dispensers at drug stores, patients attending a hospital and clinicians. Interviews were audio-recorded, translated and transcribed into English and coded using a combination of an inductive and deductive approach.

**Results:**

Drug shops were the primary location where patients engaged with health services. Interactions were brief and transactional: symptoms were described or explicit requests for specific medicine made, and money was exchanged. There were economic incentives for clients and drug stores: patients were able to save money by bypassing the formal healthcare services. Clinicians described antibiotics as easily available OTC at drug shops. Dispensing included the empirical use of broad-spectrum antibiotics, often combining multiple antibiotics, without laboratory diagnostic and drug susceptibility testing. Inappropriately short regimens (2–3 days) were also offered without follow-up. Respondents viewed OTC antibiotic as a convenient alternative to formal healthcare, the access to which was influenced by distance, time and money. Respondents also described the complexities of navigating various departments in hospitals and little confidence in the quality of formal healthcare. Clinicians and a few dispensers expressed concerns about AMR and referred to evadable policies around antibiotics use and poor enforcement of regulation.

**Conclusions:**

The findings point to the need for clear policy guidance and rigorous implementation of prescription-only antibiotics.

**Key questions**

**What is already known?**

- Previous research in Nepal has shown that around 30% to 60% of the population self-medicates.
- More than two-thirds of community pharmacies dispense antibiotics without prescription and more than one in three patients attending private pharmacies receive at least one antibiotic.
- Qualitative research can provide essential insights to improve the implementation of existing regulations and develop new strategies to address the development and spread of antimicrobial resistance.

**What are the new findings?**

- In light of their ubiquity and low costs, drug stores were the main source of antibiotics.
- Interactions between drug store staff and patients were transactional in nature: akin to visiting grocery stores, where signs and symptoms were traded for a quick remedy.
- This quick fix circumvented perceived barriers to obtaining a prescription from clinicians and boosted the commercial incentives of drug dispensers.
- High over-the-counter (OTC) use of antibiotics was also attributed to ambiguous and evadable policy.

**What do new findings imply?**

- Control and management of OTC use of antibiotics in Nepal, as in other low-income and-middle-income countries, needs a multi-pronged and comprehensive approach.
- Reinforcing formal healthcare, increasing awareness of antibiotic and AMR among patients and drug dispensers, and reforming existing policy with local and regional AMR surveillance to regulate the OTC use of antibiotics is crucial.

## Introduction

Antimicrobial resistance (AMR) is a burgeoning health threat that claims an estimated 700 000 lives every year.^1^ The human and economic cost of AMR continues to rise and globally it is projected to account for an annual 10 million deaths and economic losses of US$100 trillion by 2050.^1^ A disproportionate number of AMR-related deaths are likely to occur in Asia because of the high burden of infectious diseases, poor regulation of antimicrobials (in humans, food-animals and agricultural products), high population density and growth.^1–4^ Excessive drug pressure is one of the main drivers of AMR.^5^ Inappropriate prescribing and purchasing antimicrobials over-the-counter (OTC)—without a prescription and or medical supervision—contribute to excessive drug pressure.^3^

Among antimicrobials, antibiotics are commonly prescribed in low-income and middle-income countries (LMICs), where around 50% of patients receive at least one antibiotic,^6^ with 30%–50% of antibiotics prescribed unnecessarily in India, China and Kenya.^7^ Particularly, in LMICs, antibiotics are also bought OTC,^2 3 5^ often for common febrile illnesses, such as respiratory tract, urinary tract or gastro-enteric infections (most of which could be of viral origin).^5^ Weak formal healthcare infrastructure, a lack of health insurance, inadequate regulation and limited awareness of the risks often lead patients in LMICs to seek care from informal providers and purchase OTC medication.^2 8^ The popularity of ‘drug shops’ in LMICs is also linked to their embeddedness in the social and cultural context: customer and shop-keeper may share a common understanding of illness causation and treatment, and/or their relationship is less affected by class, power, clinical decision-making roles, knowledge and educational divides that can delineate physician–patient interactions.^9^ When seeking assistance from drug sellers, antibiotics can offer rapid symptom relief and are affordable.^3 10 11^

The private sector, mainly OTC drug shops or pharmacies, dominates Nepal’s health service: out-of-pocket payments make up nearly 70% of all health expenditure.^12 13^ Drug shops in Nepal are regulated by the Drug Act of 1978, which outlines that pharmacists, assistant pharmacists (who are registered at Pharmacy Council of Nepal) or pharmacy ‘professionals’ with minimal training can run a drug shop by registering it with the department of drug administration (DDA)—a government body to oversee the medicines, qualities and their use.^13^ Drugs in Nepal are categorised to regulate the dispensing and prescription. For instance, category ‘a’ (narcotics and poisonous drugs) and category ‘b’ (antimicrobials and hormones) can only be prescribed by a physician and dispensed by a licensed pharmacist/professional. Although category ‘a’ drugs require explicit records of their sale, category ‘b’ drugs do not require a record.^14^ In addition, drug shops are inadequately regulated, often staffed by unqualified personnel. Although there is broad acknowledgement of generic gaps in policies, regulation and implementation related to antimicrobial use, engagement with policy-makers and stakeholders regarding appropriate use of antimicrobials is inadequate.^3 4 15^

Researchers have examined various dimensions of antibiotic use and AMR in Nepal, for instance, antibiotic use in primary health care,^16^ awareness of antibiotic use and resistance in the general public^17 18^ and among students,^19^ and self-medication (with antibiotics) among nursing, dental and medical students.^20^ One study surveyed antibiotic dispensing by drug retailors in Kathmandu,^21^ but no research has comprehensively examined the drivers of OTC antibiotic sales from the perspective of the main stakeholders: patients, clinicians and dispensers. Drawing on the experiences and opinions of patients, drug dispensers and clinicians from three tertiary hospitals, this article explores the drivers of OTC antibiotic sales in western, eastern and central Nepal.

## Methods

### Study settings

**Figure 1:**
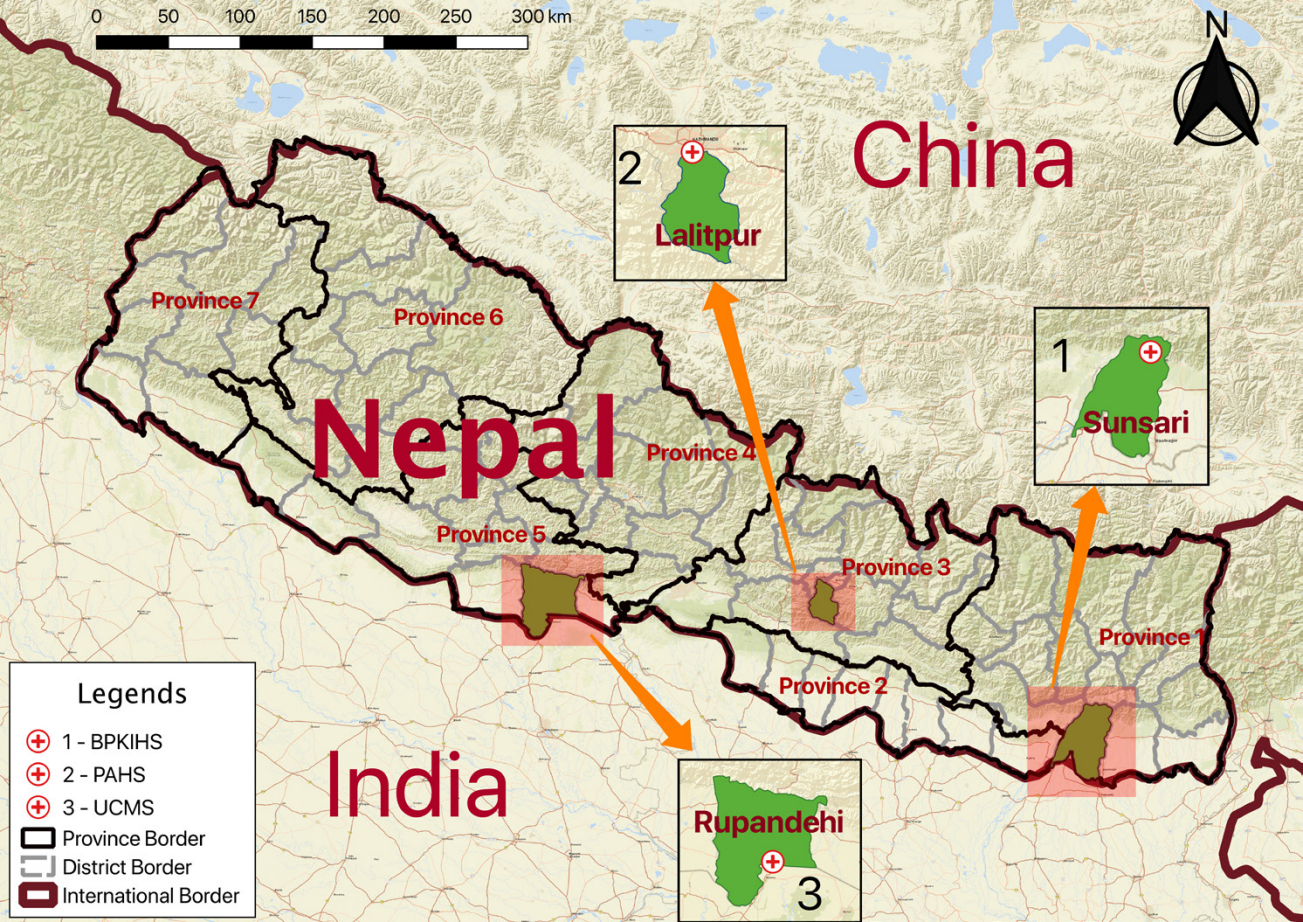
Study sites from various provinces and hospitals. The map was designed using the free and open-source GIS software: QGIS. The shape files were obtained from the Government of Nepal, Ministry of Federal Affairs and Local Development and were publicly available for unrestricted use (https://data.humdata.org/dataset/admin-shapefiles-of-nepal-mofald).

Nepal is divided into seven provinces and its health system comprises federal, provincial and local level healthcare facilities.^22^ Nepal’s public health system is hierarchical and comprises community healthcare units, primary healthcare centres, district/provincial hospitals and tertiary hospitals. In parallel, private hospitals, nursing homes, polyclinics and pharmacies serve much of the population. People seek assistance at the public and private facilities depending on the severity of illness, accessibility of services and their financial circumstances.^22^ This study was conducted in purposively selected three tertiary teaching hospitals each located in province 1 (B. P. Koirala Institute of Health Sciences/BPKIHS), province 3 (Patan Academy of Health Sciences/PAHS) and province 5 (Universal College of Medical Sciences/UCMS), and the drug shops located around these hospitals (figure 1).

### Study design

This qualitative study was conducted with patients, dispensers and clinicians and follows a standard consolidated criteria for reporting qualitative studies (COREQ) guideline (online supplemental appendix 1).^23^ Members of the research team have previously observed and interacted with dispensers and clinicians in Nepal. They have also accompanied relatives to the hospitals. Several have been posted in rural areas of Nepal as a clinician; some grew up in rural areas. The research team drew on these lived experiences to design the study, influencing the respondent types selected, recruitment procedures and data collection methods.^24 25^

10.1136/bmjgh-2021-005829.supp1 Supplementary data

*Dispensers* refer to anyone who sells antibiotics at a pharmacy/drug shop, including drug sellers (with little or no formal pharmacy training) and qualified pharmacists. Dispensers work in 'drug shops' (which is a literal translation of the Nepali: *‘Aushadi pasal’),* a term used in this article for private or public pharmacies or drug stores where antibiotics are available for purchase. Focus group discussions (FGDs) were conducted with patients and clinicians; semi-structured interviews (SSIs) were undertaken with patients, clinicians and dispensers.

### Participant recruitment

The study was co-ordinated by at least one clinician working at each site, where major tertiary hospitals were purposively selected. Patients were recruited from various departments who attended each of the tertiary hospitals. Clinicians were approached from various levels and departments. Dispensers were recruited from drug shops from around these tertiary hospitals. All participants were selected purposively for this study. Patients with experience of visiting drug shops in the past were interviewed regarding their reasons for seeking healthcare at tertiary health centres, and why and under what circumstances they visit drug shops. This allowed us to compare why people attend hospital versus drug shops. Recalling past experience has been an established method to explore the health-seeking behaviour.^26^

A total of 90 respondents from three different study sites participated in the study (online supplemental appendix 2). Respondents from various age groups and experiences were selected to draw a broad spectrum of perspectives relevant for this study. A total of eight FGDs (n=37) and five SSIs (n=5) with patients (online supplemental appendix 1, table 1), four FGDs (n=25) and eleven SSIs (n=11) with clinicians (online supplemental appendix 2, table 2), and twelve SSIs (n=12) with dispensers (online supplemental appendix 2, table 3) were conducted. Data were collected through FGDs and SSIs to garner information on individual experiences and normative practices and because it was not always possible to convene groups of respondents with busy schedules. A total of 11 out of 23 dispensers declined to participate for various reasons, mostly referring to their busy schedules. Two patients refused to participate because of severity of their illnesses, and one clinician refused to participate because of change in his schedule.

10.1136/bmjgh-2021-005829.supp2 Supplementary data

The recruitment of participants for SSIs and FGDs continued until theoretical saturation was reached, whereby no new findings emerged from subsequent interviews.^27^

### Data collection

The lead interviewers (ST and JA are male clinicians) received training in interview techniques from SP (MD, MPH) and BA (MD, DPhil), who have over 10 years’ experience of qualitative social science research. The lead interviewers conducted face-to-face SSIs and FGDs at a convenient (and quiet) location for the respondents, and were working as clinicians at these institutions during the study. For instance, most of the FGDs and SSIs with patients were conducted in the departmental meeting rooms. FGDs and SSIs with clinicians were conducted in the duty rooms within the departments. SSIs with dispensers were conducted in quiet locations behind the busy drug stores. Each of the FGD/SSI lasted between 30 and 60 min.

All FGDs and SSIs were conducted with the help of a thematic guide prepared and reviewed by BA, SP and CP (online supplemental appendices 3–5). Themes within the guides were discussed among the authors and piloted with respondents from each group (patients, dispensers and clinicians). The thematic guide was amended based on their feedback, and were adapted for SSIs and FGDs by interviewers. Following an approach previously used,^28^ thematic guide for this study outlined major themes and sample questions that allowed an interviewer to adapt and use it flexibly for SSIs and FGDs.

10.1136/bmjgh-2021-005829.supp3 Supplementary data

10.1136/bmjgh-2021-005829.supp4 Supplementary data

10.1136/bmjgh-2021-005829.supp5 Supplementary data

Oral informed consent was obtained from each participant before the FGDs and SSIs, with consent audio-recorded. All respondents were assured about the confidentiality of their responses. Brief informal discussions on their occupation were conducted to facilitate their candid expression and participation. No incentives were provided to any of these participants.

Interviewers conducted SSIs and FGDs in Nepali and audio recorded the discussion using mobile phones, in addition to writing down the field notes. No repeat interviews were conducted with any of the participants. All recorded interviews/discussions were later transcribed and translated into English by SS, RY, SP and JA. Transcripts were cross-checked by SP and lead interviewers (BA, JA and ST) against the audio files for missing utterances and subtle nuances.

### Data analysis

Thematic analysis of the transcripts was conducted in parallel with data collection by two investigators (BA and SP). None of these transcripts were shared with participants for their opinion. All transcripts were collated in NVivo for line-by-line coding. First, using a deductive approach, data were categorised based on the themes for the FGD/SSI guides; additional categories and themes were added, inductively, to incorporate the emerging themes from the transcripts.^29^ Final themes were discussed among the authors and were later categorised into main themes and sub-themes. Broad themes and sub-themes form the basis of the results section. Adhering to a principle of triangulation,^30^ a mix of balance between delineating the differences and common perspectives between three various respondents are presented under each theme. Excerpts were chosen based on their relevance to the themes in addition to the recurrences and uniqueness.

## Results

**Figure 2:**
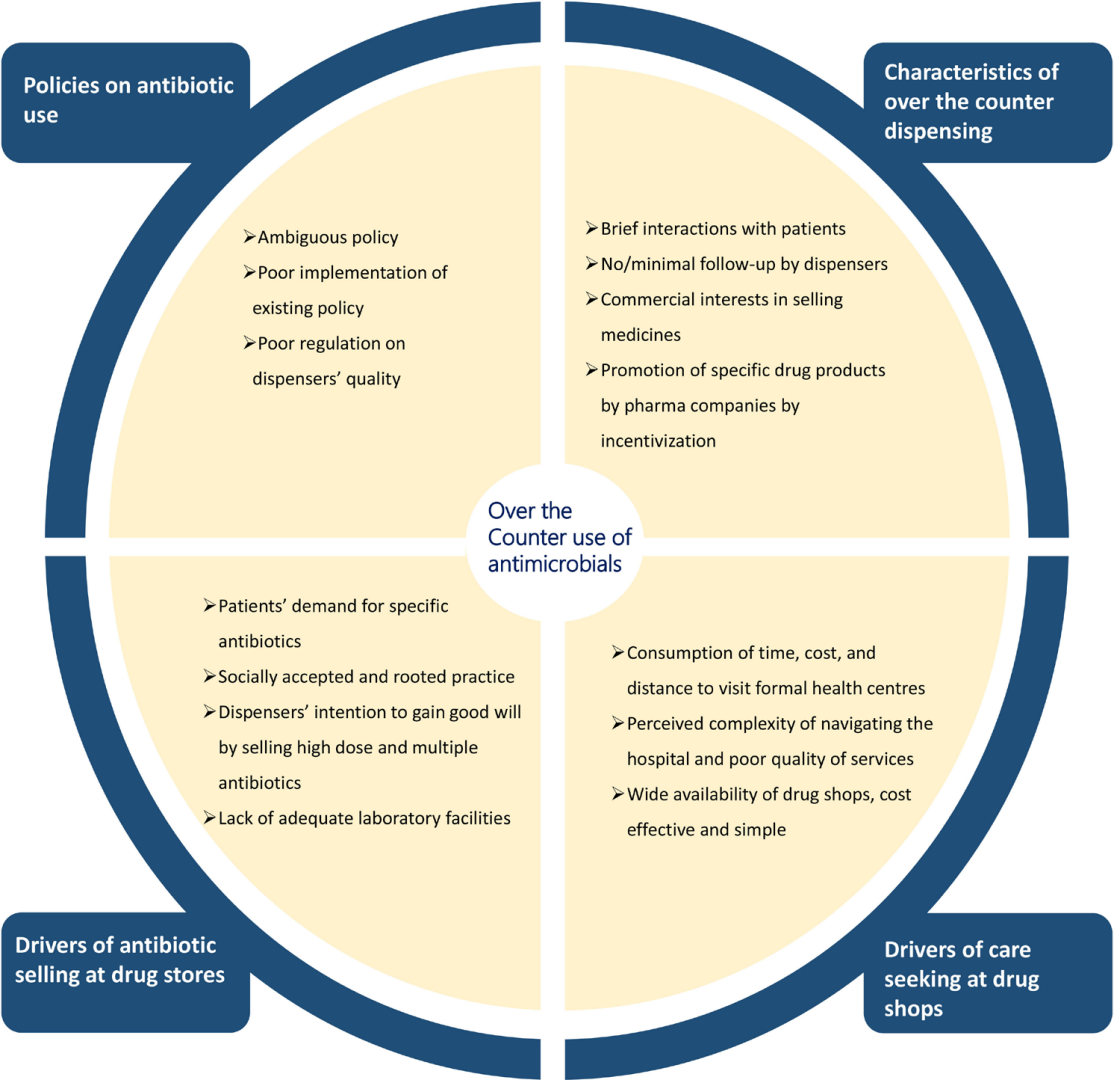
Summarised factors affecting over-the-counter use of antibiotics in Nepal.

The practice of buying medicine and antibiotics OTC depended on multiple factors, which were described by dispensers, patients and clinicians (figure 2). Dispensers were the main interface between formal and informal healthcare. In rural and urban regions, all respondents reported that drug shops were ubiquitous and constituted essential infrastructure in their community. Dispensers were aware that antibiotics were sold with little interactions with patients, that empiric treatment was common, and often entailed inappropriate dosage and overtreatment with broad-spectrum antibiotics. They were also aware that these practices contravened public health recommendations. By selling antibiotics OTC, they described how they were responding to patients’ demands and the barriers that patients face when accessing formal healthcare centres. A lack of time, costs, accessibility and perceived quality led patients to seek assistance outside formal healthcare centres. Only a few dispensers and patients were aware of AMR and described AMR as a potential consequence of inappropriate and overuse of antimicrobials through OTC. Dispensers and clinicians referred to ambiguous policies regarding the use of antibiotics, a lack of implementation of existing policies and impunity for inappropriate practice as main reasons for high OTC antibiotic sales and AMR in Nepal.

In the next section, the characteristics of OTC microbial sales, their drivers, consequences and relevant policies are described.

### The nature of OTC sales

Almost all patients described their experience of visiting drug shops for their own, family members’ or friends’ illnesses. Often the interactions were focused and brief: they resembled retail transactions, whereby medicines were purchased for specific illnesses, rather than patient consultations. Selling antibiotics for infectious diseases was one strand of everyday business in the drug shops: all dispensers explained such a business-as-usual approach and described how sometimes antibiotics were dispensed empirically when patients presented with signs and symptoms of infectious diseases. Taking no responsibility for the outcome, dispensers did not generally follow-up after selling antibiotics.

We don’t call them for follow ups. As we are not doctors, we ask them to take those medicines for certain days and ask them to visit a doctor if the medicine doesn’t work within the period (20-year-old, male, SSI-1 dispenser, Bhairahawa).

### Drivers of care-seeking at drug shops

Dispensers often described how patients hesitated to visit hospitals because of the costs, time and complexity involved:

Usually doctors ask to carry out different tests. So, I think they feel burdened to spend money on such tests and thus, they try medicines first (22-year-old, female, SSI-1 dispenser, Kathmandu).

Also, patients who travel a long distance from rural regions are poor, constrained by time and opportunity costs, and are unfamiliar with the ‘complex’ systems at the hospitals.

Clinicians echoed dispensers’ comments regarding how patients from rural regions face challenges related to visiting hospitals and communicating with doctors, particularly referring to the gaps in doctor–patient relationship, and their unfamiliarity with the hospital system.

For people from remote regions… [hospital is] a different world for them. They are scared to visit hospitals. They fear how to speak to doctors, they believe doctors are high up [educated, and superior]. There are gaps in doctor–patients’ relationships; and they do not know what to speak, how to express the problem and where to go in a hospital… all kinds of hesitations, you know (24-year-old, female, FGD-2 clinician, Bhairahawa)

All patients described visiting drug shops for mild to moderate illnesses. Direct and indirect expenses incurred in travelling to the hospitals and the cost of laboratory tests at hospitals were identified as the main barriers to seeking care at hospitals:

If you visit big hospitals they ask you to do blood tests even if you have simple fever; they don't give you medicine without test. But if you go to a pharmacy and ask for medicine worth Rs.10, it works, and it actually works. So, if Rs.10 medicine works, why go for blood test and other tests at hospital? (33-year-old, male, FGD-1 patient, Dharan).

Patients were even more fearful of the excessive costs of visiting private hospitals or clinics. Although patients did weigh other options and discuss with family members where to seek care, a nearby drug shop was often described as the most appealing, despite their confidence in the clinicians working in hospitals.

Particularly in rural areas, the poor accessibility of the formal healthcare sector was viewed as a prominent driver of patients seeking care at dispensaries. A few patients, who came from rural regions to attend the hospital, also mentioned specific conditions (eg, chronic diseases) for which they sought treatment from traditional healers whilst also visiting drug shops in parallel. Patients also expressed strong opinions about the quality of services at the hospitals. Complaints centred on waiting times, a lack of access to essential medicines and commercial interests embedded in clinicians’ practice at private clinics.

### Drivers of antibiotic selling at drug shops

Dispensers explained the OTC sales of antibiotics in terms of *‘patients’ demand’* for particular antibiotics. These demands were reportedly rooted in patients’ past experiences of cure from specific antibiotics or those of friends and family. Clinicians also reported that patients visit drug shops and make demands for specific medication using as prompts, the empty blisters/bottles, and old prescription that cured them or a family members’ illnesses.

Although antibiotics were easily purchasable, to the extent that patients could ask for specific medications, dispensers described only providing antibiotics to close social contacts. This contradiction potentially stemmed from an awareness that this practice contravened prescribing guidelines.

We give antibiotics to people who have close relation with us when they have fever (20-year-old, male, SSI-1 dispenser, Bhairahawa)

Although a few clients may demand specific antibiotics from drug shops, most of the interviewed patients did not seem to know what the term ‘antibiotic’ meant. They reported describing their symptoms to dispensers who would make decisions. Hence, patients placed their trust in dispensers to make a presumptive diagnosis. Such interactions led to repeat visits and strengthened the social relationships perpetuating the cycle of antibiotic dispensing.

Patients also described OTC as a social practice that was inherited through the generations in urban, peri-urban and rural settings. Younger generations seem to follow their parents in seeking OTC medications as a way of circumventing the treatment-seeking at hospitals or from clinicians.

I think it’s because of the ongoing practice in our society. The young generations follow their parents. They have seen their parents going to pharmacies before going to clinics/hospitals, thus they are doing the same. This practice has become a trend in our society. They are doing what they are seeing for years (42-year-old, male, SSI-2 dispenser, Kathmandu).

Clinicians were vocal about the excessive pressure from pharmaceutical companies and their representatives who visited clinics and drug shops to encourage them to prescribe particular brands of antibiotics. These pharmaceutical representatives often provide incentives (both monetary and non-monetary) and have undue influence on the prescription.

Dispensers had commercial interests in serving patients and building goodwill for future visits; they also sold the antibiotic that the pharmaceutical representatives incentivised. Patients did not explicitly acknowledge that drug shops might be motivated by profit rather than providing the most appropriate care. Although clinicians recognised that there were many drivers of OTC sales of antibiotics, including patients’ demands, few were critical about the business motives of dispensers with regard to the sale of antibiotics.

According to dispensers, there was varied expertise among the other drug shops around them. They described how, in other drug shops, sometimes an owner would assess patients and dispense antibiotics based on experience alone. The license certificates that hang on the wall of drug stores are often bought from a license holder and hold no relation to the personnel selling medicines OTC.

### Awareness of antibiotics and resistance

Dispensers were asked specifically about the antibiotics sold for infectious diseases: in most cases, they described antibiotics provided for 2 to 3 days and sometimes in sub-standard doses. Most dispensers provided broad-spectrum antibiotics including injectables to cover wide range of diseases. ‘Providing broad-spectrum antibiotics for only few days’ was considered a *‘double-edged sword’* because it could either cure a broad range of diseases, and if not cured, patients were asked to visit doctors. Patients reported that, in general, drug shops provide higher-dose medicines that were likely to cure their illnesses without having to undergo various tests at hospital, which ultimately would save their time and money. In contrast to dispensers’ claim that they were hesitant to dispense antibiotics, patients described how drug shops usually provided antibiotics easily, and in high doses.

A clinician shared his experience of how a relative received a few doses of broad-spectrum antibiotics from the drug shop, developed respiratory complications and required intensive care treatment for 19 days.

There was a patient (my relative) with 1–2 days fever who took multiple medicine from a drug shop at first and came to the hospital after 3–4 days and… suddenly had Pneumonia… and then ARDS [Acute Respiratory Distress Syndrome] and was to be admitted for 19 days (26-year-old, male, FGD-1 clinician, Bhairahawa).

Dispensers were aware of the sensitive nature of these antibiotics in terms of their judicious use based on a clinician’s prescription or confirmatory diagnosis. Some were quite vocal about the adverse implications of their potentially inappropriate presumptive treatment. However, they did not spontaneously mention the development of AMR. They described this leading to ‘*a double loss*’ if treatment did not work and the patient had to visit a formal health centre. To prevent this, dispensers were inclined to sell high-dose and newer-generation antibiotics. The dispensers also mentioned patients’ lack of compliance in completing the dose once relieved from the symptoms.

The broader adverse effects of sub-standard doses and lack of compliance were not easily translated to antibiotic resistance. Many dispensers seemed complacent about the eventual ineffectiveness of the antibiotics due to OTC use; only a few made the connection between the effectiveness of the antibiotic and resistant bacterial strains.

It means the medicine will stop working with same effectiveness later as it should. Gradually, it will stop being effective (22-year-old, female, SSI-1 dispenser, Kathmandu).

When probed, dispensers alluded to the implications of antibiotic resistance, such as a reduction in effectiveness of the antibiotics. Even if they were not aware of the mechanisms of resistance, many dispensers attributed declining effectiveness of antibiotics to patients’ incomplete adherence, and r dispensers’ or clinicians’ practice of providing high doses of antibiotics including their own practice of providing high dosage with multiple antibiotics.

Clinicians and dispensers explained the adverse consequences of prior empiric use of antibiotics, such as complexities of disease presentation (atypical presentation) and outcome (not responsive to previously effective antibiotics). Reasons and potential measures for the mitigation of the high empirical treatment with antibiotics, mostly in the peripheral region of Nepal, were fiercely debated by clinicians; the OTC practices were particularly seen as rooted in health system constraints such as lack of laboratory facilities.

Some dispensers linked rising AMR to widespread use of antibiotics in animal foods, particularly chicken. These concerns were echoed by clinicians, who further explained the misuse of antibiotics in agriculture and animal food, mostly referring these practices to how farmers themselves over-used these antibiotics.

### Policies on antibiotic use

Awareness of antibiotic prescribing policy was fairly consistent among the respondents. Dispensers explained that all medicines, except for narcotics and the medicines for mental health, could be sold at drug shops without a clinician’s prescription. A few were aware that some of the medicines that they sell need a prescription from health workers:

…[antibiotics] come under Group C so it can be sold and also it needs prescription (22-year-old, female, SSI-1 dispenser, Kathmandu).

To meet the legal requirements to dispense antibiotics, many of drug shops offer an appointment with healthcare providers, such as physician, health assistant, community medicine assistant (CMA) or auxiliary nurse midwife (ANM), who are certified for clinical decision-making to varying degrees. These clinicians generally work within the local clinics/hospitals. However, dispensers (usually a pharmacist or a non-qualified salesperson) often dispensed all available antibiotics on their behalf from drug shops without their prescription, and without taking a full history, physical examination or laboratory investigation:

If we look in most of the pharmacies, only a single person dispensing medicines is a pharmacist, whereas he is helped by CMAs or ANMs on paper. CMAs and ANMs have mostly covered the pharmacies. What shall I say; they are allowed to prescribe medicines but are not allowed to sell it [and dispensers are allowed to sell only with the prescription]? (42-year-old, male, SSI-2 dispenser, Kathmandu)

The policy that antibiotics must be prescribed by healthcare workers, who could be a CMA, but that CMAs can not not dispense the medicines promotes confusion. This ambiguous policy also obliges drug dispensers to look for some kind of legitimate approvals to display on the walls of the drug shop which equally helped them to cover up the informal drug dispensing practice. Most dispensers were quite relaxed in terms of dispensing antibiotics, whereas some were unaware of policies regulating antibiotics except for few drugs that strictly required prescriptions.

In addition to the gaps in policies around dispensing antibiotics, most clinicians targeted their frustrations at the lack of enforcement of the existing regulations, and patients’ practice of buying medications from the drug shops without a prescription.

## Discussion

In Nepal, with limited healthcare coverage and a high burden of infectious diseases,^8 31^ seeking OTC medication is a common practice.^2 3^ This is largely driven by accessibility, costs, time and the quality of services at formal healthcare centres. Drug shops offer convenient and approachable health services for patients, where poor-quality care is compensated by the minimal direct, indirect and opportunity costs, and ease of access. Clients often receive inappropriately short treatment courses (usually of 2–3 days) at drug shops and are advised to visit doctors/hospitals if not cured.

### OTC dispensing and its implications

Drug shops are a recognised part of health system infrastructure in LMICs, including Nepal.^32^ They reach a large population, offering an immediate and cost-effective service through OTC medication. Drug shops are often run by under-qualified non-professionals, such as non-pharmacists, which can lead to sub-optimal care and undesired outcomes for patients.^33^ Dispensers’ lack of follow-up and advice to visit doctors if not cured has several ramifications. This practice ostensibly encourages patients/customers to visit doctors, but it also acts as a safety net for dispensers to avoid accountability for the prognosis and the adverse outcomes of the medicine sold. If patients/customers recover, the drug shops and the drug dispensers gain easy goodwill; if not, their advice to visit doctors simply insulates them from patients’ or their familys' complaints.

Patients’ visits and interactions at drug shops resemble retail transactions.^3 33^ Patients used past medicine strips or blisters, old prescriptions, name of the medicines, and symptoms as hints to prompt pharmacists or drug dispensers to sell specific medicines. Financial incentives to dispensers also played a role in dispensing practices to the extent that particular medicines sometimes exceeded patients or customers’ needs (such as selling multiple antibiotics, stronger and expensive antibiotics). Representatives from pharmaceutical companies were recognised as playing a role here: they incentivise to increase the sale of their particular brands of drugs.^34^

Dispensers’ lack of competence was a major concern among clinicians and (other) dispensers. Most drug shops, particularly in rural Nepal, are run by unqualified personnel (non-pharmacists) who learn during their work and operate using someone else’s license (which is generally displayed on the drugstore wall) or have no license at all.^33^

### Drivers of care-seeking at drug shops and its implications

Many studies have explored access to formal healthcare services, identifying barriers such as distance, cost and perceived quality of services in Nepal and other LMIC settings.^26 35–40^ Against the backdrop of visiting drug shops and traditional healers, which are easily accessible and considered part of everyday life in rural regions, and for many who often have little education, navigating a hospital environment can be intimidating.^41^

Patients from rural regions often perceive doctors to be from a higher social class. They seem less approachable and patients find it difficult to express their problems to them. This (perceived) social distance could lead to poor doctor–patient relationships, unmet expectations and resentment.^42^ To mitigate these feelings of inferiority, patients often seek assistance from a companion when visiting hospitals, which often multiplies the costs to be borne by the patient. By seeking care in drug shops, patients can avoid the feelings of inferiority, apprehension and the cost of taking care of the companion associated with attending a hospital.^43^ In addition, often hospitals are perceived to be expensive and beyond their economic capacity, without the anticipated quality.^35^

In Nepal, drug shops began to open shortly after the establishment of the first allopathic health centre, Bir Hospital, in 1890. The number of drug shops increased greatly in the late 2000s: from around 400 in 1979 to 7000 in 1992 and ~29058 in 2021.^44^ Drug stores have hence become an important health infrastructure of modern medicine in Nepal as elsewhere.^45^

### Drivers of antibiotic selling at drug shops

The practice of providing multiple and broad-spectrum antibiotics for a wide group of illnesses echoes findings from previous studies in Nepal: antibiotics were dispensed for viral infections and self-limiting gastro-enteritis.^13 21 33 43 46^ In one study, when mock patients with dysuria and a child with watery diarrhoea presented at the drug shops, 97% of the dispensers provided unnecessary antibiotics for diarrhoea and 38% provided antibiotics for dysuria.^21^ The findings also reflect those from studies in other LMIC settings, such as Tanzania^47^ and Jordan.^48^ It is common practice to dispense antibiotics OTC with recommendations to visit doctors when not cured. This practice delays appropriate care and promotes incomplete treatment often with sub-standard dosages, potentially engendering AMR.^49^

Previous studies have highlighted antibiotic dispensing practices in drug stores,^43 50^ which must be considered within the broader social, cultural and policy context of Nepal. Studies from Nepal have consistently attributed high OTC selling of antibiotics to dispensers’ willingness to overlook the policy and ignore their own misgivings.^33 43^ This is coupled with patients’ demands for rapid relief and financial incentives to acquiesce to these demands. Dispensers have a direct financial interest in an immediate sale and a desire to maintain goodwill to ensure future sales. This resonates with findings from many LMICs, where patient demands exert pressure on dispensers.^51–53^ Especially in rural regions, high demand and consumption of antibiotics based on empiric decision were facilitated by a lack of laboratory facilities for confirmatory diagnosis.^43 54^

The empiric use of broad-spectrum antibiotics often in high doses was driven by dispensers’ intention to avert the ‘double burden’ (cost of visiting the drug shop and if not cured, the cost of visiting a hospital). The OTC use of broad-spectrum antibiotics is likely to drive AMR^5^ and the impact on AMR is compounded by patients’ sub-optimal adherence to the treatment regimen.

Patients are not aware of the potential pitfalls of purchasing medicines OTC from dispensers. They were often unaware of purchasing antibiotics. They hence almost never realised that buying these medicines OTC—without supervision—can promote AMR, reducing their future effectiveness. These findings resonate with those of previous surveys in Nepal, which reported very limited knowledge of antibiotics among non-science students^17^ and patients.^18^ There was little awareness of the impact of inappropriate antibiotic use among other respondents: only few dispensers knew about its potential consequences in terms of AMR. Dispensers were not explicit about AMR but a few explained an impact in terms of possible decline in effectiveness of antibiotics if overused. This reflects the limited knowledge about antibiotics and AMR in the general population, which is commonly reported in Nepal^17 19^ and elsewhere.^55 56^ Many dispensers struggled to explain what AMR was and its contributors, although sometimes associations were made with patients’ poor adherence to drugs and the dispensing of high-dose, broad-spectrum and multiple antibiotics. Also, a few dispensers (without prompts) and clinicians signalled the widespread use of antimicrobials in food animals and agricultural products: a reference to the fact that strategies to counteract OTC and AMR require an inclusive ‘one health approach’.^2–4^ The shared experience of clinicians and dispensers about treatment failure with regular antibiotics and their evolving practice in selecting antibiotics signalled that the AMR was already influencing clinical practice.

### Implications for health policy and planning

Only a few dispensers and clinicians had specific knowledge of policies regarding antibiotics. Policies addressing the widespread use/misuse of antibiotics OTC in Nepal were regarded ambiguous and poorly enforced. Specifically, lack of stringent policies regulating dispensing of antibiotics, and ambiguity in prescription versus dispensing require urgent attention. Clinicians also reflected on the lack of adequate health infrastructure and the essential nature of antibiotics in terms of preventing deaths due to infectious diseases. This resonates with the broader recognition that antibiotics are often crutches for fractured health system infrastructure, poverty, poor water and sanitation, and high burden of infectious diseases.^2 57 58^ Thus, policies around antibiotic use and their implementation is paramount for judicious investment in building robust health system with universal health coverage.^59^ Restricting OTC use of antibiotics alone without universal health coverage may have adverse consequences.^60^ Because the bulk of the antibiotics are dispensed through the drug shops, providing regular training to the dispensers, for instance, through the stewardship of local and regional health authorities together with the broader community and stakeholder engagement, is critical to reduce inappropriate demands for antibiotics and facilitate the good practice in antibiotic dispensing.^61^

The lack of knowledge about antibiotics among patients, and inadequate knowledge about the adverse consequences of OTC antibiotics and AMR among dispensers has critical implications for national policies and strategies. Community and stakeholder engagement strategies^62–64^ are crucial to ensure these specific population receive tailored and comprehensive training and awareness on antibiotics, its use (OTC) and the adverse consequences. For instance, nationwide community engagement strategies are needed to ensure community members understand what antibiotics are and to foster awareness of how (over and inappropriate) consumption can lead to AMR.

### Strengths and limitations

This is the first study to explore the perspectives from three most relevant stakeholders (patients, clinicians and the dispensers) on the OTC use of antibiotics from tertiary hospitals located in different provinces of Nepal. OTC practices were compared across these regions: respondents from urban and rural regions described high OTC use of medications combined with particularly poor quality of care in the rural regions. Because of limited health services, including the drug shops in rural regions, patients reported a reliance on local health services/drug shops before deciding to travel in search of quality healthcare and reflects other evidence that indicates the urban–rural divide in health services across Nepal.^22 35 36 65–67^ To maximise the reliability of findings, the perspectives of stakeholders with varied and conflicting interests were collected and triangulated.^30^ Collecting data through a mix of SSIs and FGDs is likely to have compensated the limitations of the individual method.

Approaching dispensers for interview was particularly difficult because of their hesitance around revealing their practice of selling antibiotics. During SSIs, they often began cautiously and the interviewer was forced to ask about dispensing practices indirectly to probe further their perception and practices. Although interviews were conducted in a way to build rapport, using non-leading questions and responding in a non-judgemental manner, power difference between clinicians and patients may have engendered social desirability bias.

This study focused on human health alone and did not address the widespread use of antimicrobials in livestock and agricultural products. Future research embracing ‘One health approach’ would be useful to examine attitudes to antibiotic use and the development of resistant infections among livestocks.

## Conclusions

Purchasing antibiotics OTC is an established practice in Nepal with many drivers. Faced with constraints of money, time and distance, the complexities of navigating services in hospitals, and intimidating social hierarchies, patients often visit drug stores. When in drug stores, decisions to dispense antibiotics—often in the absence of laboratory investigations—were influenced by patient demand (driven by a preference for rapid symptom relief) and dispensers’ intention to gain goodwill. Antibiotic doses were often inappropriate and there was a perceived lack of compliance and follow-up. Current national policy on OTC purchasing of antibiotics was recognised as ambiguous and poorly enforced. Policies to address inappropriate antibiotic use and AMR should be embedded in a broader drive to improve health infrastructure. Specific AMR-related initiatives must be multipronged: re-allocating the resources in rural regions to scale up the quality health services, strengthening antimicrobial resistance surveillance, and stakeholder and community engagement, and awareness.

## Data Availability

Data are available on request. Data cannot be shared publicly because of the nature of the data being qualitative that contains personal quotes and clues to where the study occurred and can be potentially identifiable. However, anonymised data are available on request to the research department complying with the data access policy outlined by Institutional Review Committee (IRC) of Manmohan Memorial Institute of Health Sciences (https://www.mmihs.edu.np/irc.php).
