# Supplementary material for: Why do people purchase antibiotics over-the-counter? A qualitative study with patients, clinicians and dispensers in central, eastern and western Nepal
Source: BMJ Glob Health. 2021 May 11;6(5):e005829. doi: 10.1136/bmjgh-2021-005829 (PMC8118002; doi:10.1136/bmjgh-2021-005829)
Supplement: Supplementary data [file bmjgh-2021-005829supp002.pdf]

Appendix 2: Socio-demographic details of respondents

**Table 1: FGDs and SSIs with patients**						
**Data type**	**Age**	**Sex**	**Education in years**	**Occupation**	**Hospital**	**Location**
**FGD Bhairahawa**						
FGD_BP-1	46	Male	10	Farmer	Surgical ward/UCMS	Bhairahawa, Province 5
	36	Female	none	Farmer		
	33	Male	9	Sweets seller		
	27	Female	5	Housewife		
	61	Female	none	Housewife		
FGD_BP-2	21	Female	12	Student	Medical ward/UCMS	Bhairahawa, Province 5
	40	Female	8	Housewife		
	21	Male	9	Civil service		
	45	Male	2	Farmer		
FGD_BP-3	42	M	8	Ex-army	Medical ward/UCMS	Bhairahawa, Province 5
	36	F	8	Housewife		
	40	F	None	Laborer		
	57	M	3	Technician		
	40	M	10	Driving		
	35	M	5	Construction worker		
FGD_BP-4	60	M	10	Laborer	Medical ward/UCMS	Bhairahawa Province 5
	40	F	None	Farmer		
	52	F	6	Farmer		
	74	M	7	Farmer		
	56	M	6	Farmer		
	24	M	10	Technician		
FGD_BP-5	38	M	8	Driving	Medical ward/UCMS	Bhairahawa, Province 5
	21	F	10	Housewife		
	22	M	10	Clerk		
	28	F	10	Housewife		
	30	M	5	Technician		
**FGD Dharan**						
FGD_DP-1	30	Male	CA	Banker	Various wards/BPKIHS	Dharan, Province 1
	33	Male	SLC	Business		
	32	Male	8	Farmer		
	42	Male	4	Farmer		

FGD_DP-2	36	Male	SLC	Businessman	Various wards/BPKIHS	Dharan, Province 1
	56	Female	7	Housewife		
	27	Female	8	Housewife		
	40	Female	SLC	Housewife		
FGD_DP-3	35	M	3	Farmer	Various wards/BPKIHS	Dharan, Province 1
	43	M	9	Business		
	28	M	5	Laborer		
**SSI Kathmandu**						
SSI_KP-1	44 years	Male	12	Social service	Patan hospital	Lalitpur, Bagmati
SSI_KP-2	52	M	SLC	Driver	Patan hospital	Lalitpur, Bagmati
SSI_KP-3	31	F	Undergraduate	Accountant	Patan hospital	Lalitpur, Bagmati
SSI_KP-4	44	M	Bachelor in Arts	Businessman	Patan hospital	Lalitpur, Bagmati
SSI_KP-5	51	F	SLC	Housewife	Patan hospital	Lalitpur, Bagmati

Table 2: FGDs and SSIs with clinicians

**Data type**	**Age**	**Sex**	**Education in years**	**Occupation**	**Hospital**	**Location**
**FGD-Bhairahwa**						
FGD_BC-1	25	M	Intern	Intern Physician	UCMS	Bhairahawa, Province 5
	26	M	Intern	Intern Physician		
	25	M	Intern	Intern Physician		
	26	M	Intern	Intern Physician		
	25	M	Intern	Intern Physician		
FGD_BC-2	25	F	Intern	Intern Physician	UCMS	Bhairahawa, Province 5
	24	F	Intern	Intern Physician		
	25	F	Intern	Intern Physician		
	26	F	Intern	Intern Physician		
SSI_BC-1	25	M	Intern	Intern Physician	UCMS	Bhairahawa, Province 5
SSI_BC-2	26	M	Medical Officer	Physician	ER/UCMS	Bhairahawa, Province 5

SSI_BC-3	32	M	Resident Physician, second year	Physician	Medicine Department /UCMS	Bhairahawa , Province 5
SSI_BC-4	26	M	Medical Officer	Physician	ER	Bhairahawa , Province 5
SSI_BC-5	32	M	Resident physician	Physician	Medicine	Bhairahawa , Province 5
SSI_BC-6	34	M	Consultant , Internal Medicine	Internist	Medicine Department /UCMS	Bhairahawa , Province 5
**Dharan**						
FGD_DC-1	25	M	Intern	Intern Physician	BPKIHS	Dharan Province 1
	25	M	Intern	Intern Physician		
	26	M	Intern	Intern Physician		
	26	M	Intern	Intern Physician		
FGD_DC-2	25	F	Clinical Students	Final year MBBS	BPKIHS	Dharan Province 1
	24	F	Clinical Students	Final year MBBS		
	25	F	Clinical Students	Final year MBBS		
	26	F	Clinical Students	Final year MBBS		
FGD_DC-3	25	M	Clinical Students	Final year MBBS	BPKIHS	Dharan Province 1
	24	M	Clinical Students	Final year MBBS		
	23	M	Clinical Students	Final year MBBS		
	24	M	Clinical Students	Final year MBBS		
FGD_DC-4	23	M	Clinical Students	Final year MBBS	BPKIHS	Dharan Province 1
	24	M	Clinical Students	Final year MBBS		
	24	M	Clinical Students	Final year MBBS		
	26	M	Clinical Students	Final year MBBS		
**Kathmandu**						
SSI_KC-1	36	M	MBBS	Physician	PAHS	Lalitpur, Bagmati

SSI_KC-2	24	F	MBBS	Physician	PAHS	Lalitpur, Bagmati
SSI_KC-3	29	M	MBBS	Physician	PAHS	Lalitpur, Bagmati
SSI_KC-4	26	M	MBBS	Physician	PAHS	Lalitpur, Bagmati
SSI_KC-5	24	F	MBBS	Physician	PAHS	Lalitpur, Bagmati

Table 3: FGDs and SSIs with dispensers

**Data type**	**Age**	**Sex**	**Education in years**	**Occupation**	**Hospital**	**Location**
**SSI-Dispenser-Bhairahawa**						
SSI_BD-1	20	M	SLC	Dispenser	Private pharmacy	Bhairahawa, Province 5
SSI_BD-2	69	M	Bachelor in Arts	Owner of Pharmacy	Private pharmacy	Bhairahawa, Province 5
SSI_BD-3	21	M	SLC	Dispenser	Private pharmacy	Bhairahawa, Province 5
SSI_BD-4	49	M	SLC_CMA	Dispenser	UCMS Hospital pharmacy	Bhairahawa, Province 5
**SSI-Dispenser-Dharan**						
SSI_DD-1	38	M	10+2 Passed	Pharmacy owner	Private pharmacy	Dharan Province 1
SSI_DD-2	69	M	SLC	Pharmacy owner	Private pharmacy	Dharan Province 1
SSI_DD-3	26	M	HA	Dispenser	Private pharmacy	Dharan Province 1
**SSI-Dispenser-Kathmandu**						
SSI_KD-1	22	F	CMA	Dispenser	Private pharmacy	Lalitpur, Province 3
SSI_KD-2	42	M	Intermediate	Pharmacy owner	Private pharmacy	Lalitpur, Province 3
SSI_KD-3	44	M	CMA	Dispenser	Private pharmacy	Lalitpur, Province 3
SSI_KD-4	42	F	Intermediate	Dispenser	Private pharmacy	Lalitpur, Province 3
SSI_KD-5	23	M	10+2	Dispenser	Private pharmacy	Lalitpur, Province 3
