# Supplementary material for: Why do people purchase antibiotics over-the-counter? A qualitative study with patients, clinicians and dispensers in central, eastern and western Nepal
Source: BMJ Glob Health. 2021 May 11;6(5):e005829. doi: 10.1136/bmjgh-2021-005829 (PMC8118002; doi:10.1136/bmjgh-2021-005829)
Supplement: Supplementary data [file bmjgh-2021-005829supp003.pdf]

Interview guide for clinicians /1

FGD/SSI number:	Location:

People present at interview:

- Use this interview guide for interviews with clinicians or medical practitioners at any of the tertiary hospitals
- Take notes during or after the interview for each topic – just the main points (in English)
- Include description of general ambience, non-verbal reactions and the rough degree of their emotions on particular topics
- You can change the questions but ensure that each issue is covered.
- Avoid using closed questions and probe frequently.
- **Obtain demographic data in the sheet below.**

**Focused Group Discussion Note Takers Sheet**						
**Note Takers initials: E.g. JA**						
**Date: DD-MM-YYY**						
**Address:**						
**Total participants numbers:**						
**Socio-demographics of participants**						
**S.N.**	**Age**	**Sex**	**Education (years)**	**Occupation**	**Remarks**	**Individual ID**
**1.**						
**2.**						

Interview guide for clinicians /2

**3.**						
**4.**						
**5.**						
**6.**						
**7.**						
**8.**						
**9.**						
**10.**						

-

1) Explain the study and ask for consent (memorise the verbal consent script as best you can), then record consent when you turn on microphone

e.g "Thank you for seeing me today…are you happy to take part in this study?

Turn the recorder on

Ok, so I have turned the microphone on. I just wanted to ask again, are you happy to take part in this study by speaking with me today?

2) Explore the themes below:

**OTC: (Definition: Buying "over the counter medicine" means purchasing medicine from a local dispenser who is not the health worker/physician or buying medicine without a prescription)**
In your opinion, what does it mean by buying medicine over the counter?<br>• Where did you learn about this?

- Can you tell us what are the legal provisions about over the counter medications?
- How do you treat patients in your everyday practice?
- Have you seen patients coming with the history of over the counter medications?
- What sort of OTC medications have been frequent in your experience?
- Have you seen the difference in patients who had OTC medications versus those who had not in your practice? (for e.g. diagnosis, treatment outcome, severity..)
- In your experience, what kind of patients in general have prior history of OTC medications?
- Do you provide specific counselling on OTC medications? If so what are they?

In your experience, what can you buy at local medicine shop?

- Does buying over the counter include buying antibiotics?
- What sort of antibiotics?
- Can they also buy injectable antibiotics?

If you/anyone buys medicine over the counter, in general what medicine do you/they buy over the counter?

Interview guide for clinicians /4

- Have you seen/experienced your relatives/colleagues (professional versus non-professional) buying medicine over the counter?

What is your opinion on effectiveness of the medicine you buy at the medical shops? / Can you tell me what do you do if OTC does not work? For how long do you usually wait for recovery? Where do you then go for treatment?

Can you also buy antibiotics at the local dispensers? (if so, can you tell me more if you need prescription to buy it?)

Do you know the consequences of buying medicine over the counter? (for example, look for if they know the antibiotic resistance, probe for buying antibiotics and its adverse consequences)<br>• What do you know about AMR or antibiotic resistance?/how is it caused?<br>• Are you aware of specific types of AMR and what are the most common in your clinical practice?<br>• What can be done to mitigate this problem?<br>• Have you come across cases of antibiotic resistance or AMR?<br>• Where did they learn about AMR/antibiotic resistance?<br>• What can be done to promote awareness of AMR?
**CLOSING**
Do you have any questions or concerns you'd like to raise? Thank you.
