# Supplementary material for: Why do people purchase antibiotics over-the-counter? A qualitative study with patients, clinicians and dispensers in central, eastern and western Nepal
Source: BMJ Glob Health. 2021 May 11;6(5):e005829. doi: 10.1136/bmjgh-2021-005829 (PMC8118002; doi:10.1136/bmjgh-2021-005829)
Supplement: Supplementary data [file bmjgh-2021-005829supp004.pdf]

Interview guide for dispensers /1

**FGD/SSI number:**	**Location:**

People present at interview:

- Use this interview guide for interviews with dispensers around any of the 3 tertiary hospitals
- Take notes during or after the interview for each topic – just the main points (in English)
- Include description of general ambience, non-verbal reactions and the rough degree of their emotions on particular topics
- You can change the questions but ensure that each issue is covered.
- Avoid using closed questions and probe frequently.
- **Obtain demographic data in the sheet below.**

**SSI Note Takers Sheet**						
**Note Takers initials: E.g. JA**						
**Date: DD-MM-YYY**						
**Address:**						
**Socio-demographics of participants**						
**S.N.**	**Age**	**Sex**	**Education (years)**	**Occupation**	**Remarks**	**Individual ID**

-

1) Explain the study and ask for consent (memorise the verbal consent script as best you can), then record consent when you turn on microphone

e.g "Thank you for seeing me today…are you happy to take part in this study?

Interview guide for dispensers /2

Turn the recorder on

Ok, so I have turned the microphone on. I just wanted to ask again, are you happy to take part in this study by speaking with me today?

2) Explore the themes below:

**OTC: (Definition: Buying "over the counter medicine" means purchasing medicine from a local dispenser who is not the health worker/physician or buying medicine without a prescription)**
What are the main illnesses that you treat (without referring to OTC)?<br>• How many customer do you see in a day?<br>• Is the place yours or do you work for someone?<br>• How long have you been working here?<br>• How long has the shop been open?<br>• Have you seen any changes in this time? What are they?<br><br>In general, how do you understand about buying medicine over the counter? In your opinion, what are its potential benefits and disadvantages?

In general, what are the medication patient spontaneously demand? What is your general reaction to them?

- Do different types of people ask for different medicines?
- For example, when a patient visits you with fever, can you tell us what do you do? (probe: do you do lab examination? Do you consult with someone else? Do you prescribe antibiotics? On what basis? when do you call for the follow up?) do patients with fever demand antibiotics?/if so, what do you do?
- What about common cold/flu, how do you treat them? Do these patients with flu demand for anitbiotics? If so, what do you do?
- Who asks for what?
- Do they directly ask for a medicine or is there a more subtle way of asking…e.g. telling a story about friend who was sick and received a certain medicine?
- Do you ever ask advice from anyone if you receive these requests?

In your opinion, what do you think motivations of buying medications amongst patients who attend your dispensary?

Can they buy antibiotics at your place? (How often do these patients come, a rough estimate? How do you deal with it? E.g. ask prescription, ask to visit a doctor, you treat based on your knowledge….)

- What types of antibiotics?
- Do you sell them as individual tablets or as a whole course?
- How much do they cost?
- Which antibiotic do you sell the most?
- What complaints are they sold for?
- Do you ever refuse to sell antibiotics?
- If you don't have them in stock can you get them from somewhere else?
- Do you ever refer customers to other providers?

Do you know the consequences of buying medicine over the counter? (for example, look for if they know the antibiotic resistance, probe for buying antibiotics and its adverse consequences)

- Have you heard about antibiotic resistance? What do you know about it? What causes it? How did you learn about it?
- Have you come across cases of antibiotics not working? What happened? Have you heard about other types of drug resistance? Can you give me some examples?

Can you tell us what are the legally sellable drugs?

- What is the legal provision for selling antibiotics with or without prescription?
- Can all dispensers sell antibiotics?
- What are the types of antibiotics that can be sold (injectable versus oral)?

**CLOSING**
Do you have any questions or concerns you'd like to raise? Thank you.
