# Supplementary material for: Why do people purchase antibiotics over-the-counter? A qualitative study with patients, clinicians and dispensers in central, eastern and western Nepal
Source: BMJ Glob Health. 2021 May 11;6(5):e005829. doi: 10.1136/bmjgh-2021-005829 (PMC8118002; doi:10.1136/bmjgh-2021-005829)
Supplement: Supplementary data [file bmjgh-2021-005829supp005.pdf]

Interview guide for patients /1

FGD/SSI number:	Location:

People present at interview:

- Use this interview guide for FGDs with patients attending General OPD at any of the tertiary hospitals
- Take notes during or after the interview for each topic – just the main points (in English)
- Include description of general ambience, non-verbal reactions and the rough degree of their emotions on particular topics
- You can change the questions but ensure that each issue is covered.
- Avoid using closed questions and probe frequently.
- **Obtain demographic data in the sheet below.**

**Focused Group Discussion Note Takers Sheet**						
**Note Takers initials: E.g. JA**						
**Date: DD-MM-YYY**						
**Address:**						
**Total participants numbers:**						
**Socio-demographics of participants**						
**S.N.**	**Age**	**Sex**	**Education (years)**	**Occupation**	**Remarks**	**Individual ID**
**1.**						
**2.**						

Interview guide for patients /2

**3.**						
**4.**						
**5.**						
**6.**						
**7.**						
**8.**						
**9.**						
**10.**						

-

1) Explain the study and ask for consent (memorise the verbal consent script as best you can), then record consent when you turn on microphone

e.g "Thank you for seeing me today…are you happy to take part in this study?

Turn the recorder on

Ok, so I have turned the microphone on. I just wanted to ask again, are you happy to take part in this study by speaking with me today?

2) Explore the themes below:

**Recent health**
How have you been in the last few weeks?

What are the health problems affecting you?<br><br>What are the common health problems in your community?<br><br>What do you mostly suffer from?
**Health-seeking behaviour**
How do you decide where to go when you are sick? (for example, severity of the illness, recognition of the illness, availability of money, transport, for old, for children-does it vary etc)

Have you got fever in the past?

- How do you know when you have fever?
- When was the last time you had fever?

When you have fever, what do you do? (probe: self-treatment, visiting traditional healers, visiting pharmacies, visiting clinics, and hospital)

If you have fever, where do you usually go first, for treatment? (if not where do you go? What are the normal course of your visits?)

Do you go to traditional healers as well for treatment?

- If so what are the health conditions that make you to visit them?

• Can you tell us if you know different kinds of traditional healers and when you visit them?<br><br>Do you go to nearby medical shop to buy medicine if you have fever? (if so why/ if no why?)
**OTC: (Definition: Buying "over the counter medicine" means purchasing medicine from a local dispenser who is not the health worker/physician or buying medicine without a prescription)**
Can you tell me how/what you understand about buying medicine over the counter?<br><br>In general, what can you buy at local medicine shop?

If you/anyone buys medicine over the counter, in general what medicine do you buy over the counter?

Can you tell us why people generally visit medical shops when they have febrile illnesses?

- Probe: reasons such as money, distance, lack of time, road conditions, beliefs, recommendations by friends, relatives…

What is your opinion on effectiveness of the medicine you buy at the medical shops?

- Can you tell us what are the different types of medicine you receive from local medical shop?
- How does the medicine compare to the ones that you receive from health facilities or the hospital?
- Do the staff at the shops give instructions on how to use the medicines? Do the packets have instructions?

Can you tell me what do you do if OTC does not work?

- For how long do you usually wait for recovery? Where do you then go for treatment?

Can you also buy antibiotics at the local dispensers? (if so, can you tell me more if you need prescription to buy it?)

- What types of antibiotics?
- Do you know their names?
- Do some shops sell and others not? Which ones?
- How much do they cost?
- Do you buy a whole packet or do they sell them one pill at a time?
- Do you take all the tablets that they give you?
- Have you ever requested antibiotics and been refused? Did they explain why?

Do you know the consequences of buying medicine over the counter?

- Do you know if overuse/use of antibiotics can contribute to antibiotic resistance?

  Do you know any other adverse consequences of buying medicine over the counter? (economic, visiting other places such as hospitals)

Do you know if legally anyone can buy antibiotics from the medical shops? If so what do you know?

Do you know any consequences of buying medicine over the counter? What about antibiotics?

Have you heard of antibiotic resistance? Can you tell us what does it mean?

Do you know any danger of taking antibiotics?

Have you heard anything about using antibiotics? Where did you hear about it?

CLOSING

Do you have any questions or concerns you'd like to raise? Thank you.
